# F177. THALAMIC SUBREGION RESTING STATE CONNECTIVITY IN SCHIZOPHRENIA MEASURED AT 7T

**DOI:** 10.1093/schbul/sby017.708

**Published:** 2018-04-01

**Authors:** Jun Hua, Ann Choe, Nicholas Blair, Anita Barber, Allison Brandt, James Pekar, Peter van Zijl, Christopher Ross, Issel Lim, Feng Xu, Russell Margolis

**Affiliations:** 1 Johns Hopkins University, Kennedy Krieger Institute; 2 Johns Hopkins University

## Abstract

**Background:**

The thalamus, a critical node in which multiple cerebral circuits converge, is organized into multiple subnuclei, classified as either first-order (receiving peripheral sensory input) or higher-order (receiving input primarily from the cortex). Higher-order nuclei are of particular salience in psychotic disorders, as they appear to control cortico-cortical information transmission, possibly through regulation of neuronal synchrony. Substantial evidence has demonstrated abnormalities of thalamo-cortical connectivity in schizophrenia, generally with hyperconnectivity with sensorimotor and temporal cortices and hypoconnectivity with frontal regions. We took advantage of the spatial resolution of 7T (voxels of 3.375 mm3 vs 27 mm3 at 3T) to preliminarily assess resting state connectivity between specific thalamic nuclei and cortical regions in schizophrenia.

**Methods:**

Resting state fMRI scans were obtained for 14 SCZ patients (mean age 39.5, mean disease duration 18.8 years) and 14 matched controls (smoking, age, sex) using a Phillps 7T imaging system, with GRE EPI (TR/TE/FA=2000/22ms/60º, voxel=2.5mm iso, 54 slices, 7min. Data analysis was carried out with SPM8 / Matlab6. Preprocessing included realignment, slice time correction, co-registration, segmentation, normalization; nuisance removal (CompCor), regression of global mean and motion parameters; spatially smoothing (5mm kernel) and temporal filtering (0.01-0.1Hz). Seed-based analysis was carried out using thalamic sub-regions as described in the Oxford Thalamic Connectivity Atlas (seven regions based on anatomic connectivity rather than histology), and whole brain connectivity maps (z values) to each seed were calculated. Second-level t-tests were performed to examine differential connectivity between SCZ patients and controls (thresholded at a voxel-level of p<.001 and multiple-comparisons corrected at a cluster-level threshold of p<.05). Effect size was estimated with Cohen’s d. The IBASPM116 atlas was used to identify anatomical regions within the significant clusters.

**Results:**

Both reduced and enhanced functional connectivity between the thalamus and multiple brain regions were observed. Statistically significant differences between scz and controls were detected in 47 regions, with particularly strong differences between scz and control in thalamo-temporal cortex connectivity, consistent with previous results at 3T. The same analysis was performed but with seeds placed in each of the seven thalamic subregions defined by the Oxford thalamic connectivity atlas. Enhanced connectivity was observed between all thalamic sub-regions and the motor cortex. Enhanced connectivity to the temporal cortex was detected in several thalamic sub-regions, but not sub-region 7, which has the highest anatomical connection probability in controls. Reduced functional connectivity in SCZ was detected between thalamic sub-regions 4, 6, and 7, and prefrontal and cingulate cortex.

**Discussion:**

Our results provide preliminary evidence of changes in resting state thalamo-cortical connectivity in schizophrenia that are specific to particular thalamic subregions. If confirmed by larger scale studies, identifying altered functional connectivity patterns of specific thalamic subnuclei may provide important clues about the pathogenic process in schizophrenia, with the potential of serving as biomarkers for use in therapeutic development.

